# 3C protease of enterovirus 71 cleaves promyelocytic leukemia protein and impairs PML-NBs production

**DOI:** 10.1186/s12985-021-01725-7

**Published:** 2021-12-20

**Authors:** Zhuoran Li, Ya’ni Wu, Hui Li, Wenqian Li, Juan Tan, Wentao Qiao

**Affiliations:** Key Laboratory of Molecular Microbiology and Technology, Ministry of Education, College of Life Sciences, Nankai University, Tianjin, 300071 China

**Keywords:** Enterovirus 71 (EV71), 3C protease, Promyelocytic leukemia (PML), PML nuclear bodies (PML-NBs), Cleavage

## Abstract

**Background:**

Enterovirus 71 (EV71) usually infects infants causing hand-foot-mouth disease (HFMD), even fatal neurological disease like aseptic meningitis. Effective drug for preventing and treating EV71 infection is unavailable currently. EV71 3C mediated the cleavage of many proteins and played an important role in viral inhibiting host innate immunity. Promyelocytic leukemia (PML) protein, the primary organizer of PML nuclear bodies (PML-NBs), can be induced by interferon and is involved in antiviral activity. PML inhibits EV71 replication, and EV71 infection reduces PML expression, but the molecular mechanism is unclear.

**Methods:**

The cleavage of PMLIII and IV was confirmed by co-transfection of EV71 3C protease and PML. The detailed cleavage sites were evaluated further by constructing the Q to A mutant of PML. PML knockout cells were infected with EV71 to identify the effect of cleavage on EV71 replication. Immunofluorescence analysis to examine the interference of EV71 3C on the formation of PML-NBs.

**Results:**

EV71 3C directly cleaved PMLIII and IV. Furthermore, 3C cleaved PMLIV at the sites of Q430–A431 and Q444–S445 through its protease activity. Overexpression of PMLIV Q430A/Q444A variant exhibited stronger antiviral potential than the wild type. PMLIV Q430A/Q444A formed normal nuclear bodies that were not affected by 3C, suggesting that 3C may impair PML-NBs production via PMLIV cleavage and counter its antiviral activities. PML, especially PMLIV, which sequesters viral proteins in PML-NBs and inhibits viral production, is a novel target of EV71 3C cleavage.

**Conclusions:**

EV71 3C cleaves PMLIV at Q430–A431 and Q444–S445. Cleavage reduces the antiviral function of PML and decomposes the formation of PML-NBs, which is conducive to virus replication.

## Background

Enterovirus 71 (EV71) is a member of the genus of *Enterovirus* of the *Picornaviridae* family [1]. It was initially isolated from patients suffering from central nervous system diseases in California in the 1960s, which, subsequently, was identified to be associated with hand-foot-mouth disease (HFMD) and aseptic meningitis [2]. Since its discovery, EV71 has caused many outbreaks and epidemics in the world [3–5], particularly in the Asia–Pacific countries, such as China, South Korea, Singapore, Japan, and Vietnam [6]. EV71 usually infects infants the age of five and causes hand foot mouth disease. The most common symptom is lesions on the skin of the hands, feet, mouth, and buttocks. A few children have cardiopulmonary dysfunction. Severe infection can lead to aseptic meningitis, brainstem encephalitis, and other nervous system diseases [7, 8]. There is currently no effective drug for preventing and treating EV71 infection [9]. The study on the pathogenic mechanism of the EV71 is important for developing anti-virus drugs and vaccines to protect the health of infants and young children.

EV71 is a non-enveloped virus with a single-stranded, positive-sensed RNA genome of approximately 7.5 kb [10, 11]. It contains one open reading frame (ORF) and encodes a single polyprotein precursor, which is subsequently split into four structural (VP1, VP2, VP3, and VP4) and seven nonstructural (2A, 2B, 2C, 3A, 3B, 3C, and 3D) proteins [12]. The 3C protein participates in many cellular processes and viral replication [13]. Three amino acid sites, His40, Glu71, and Cys147, constitute the catalytic activity core of EV71 3C, which shows serine protease and cysteine protease activities [14]. 3C also exhibits RNA binding activity [15]. Studies have shown that EV71 3C mediated the cleavage of many proteins in the host cell, including mRNA cleavage stimulation factor CstF-64, which represses cellular polyadenylation [16], telomere binding protein PinX1, which inhibits EV71-induced apoptosis [17], nuclear RNA- and DNA-binding protein TDP-43, which caused its translocation [18], etc. During viral infection, 3C protease also inhibits host innate immunity. 3C can inhibit interferon production by downregulating miR-526a, a kind of micro RNA that positively regulates virus-triggered IFN-I production, or inhibiting interferon regulation factor 7 to block the RIG-I signaling pathway [19, 20]. Furthermore, 3C can prevent inflammasome formation by degrading NLRP3 protein [21]. Considering its important role in virus life, targeting 3C protein has become a potential way to combat EV71 infection.

**Fig. 1:**
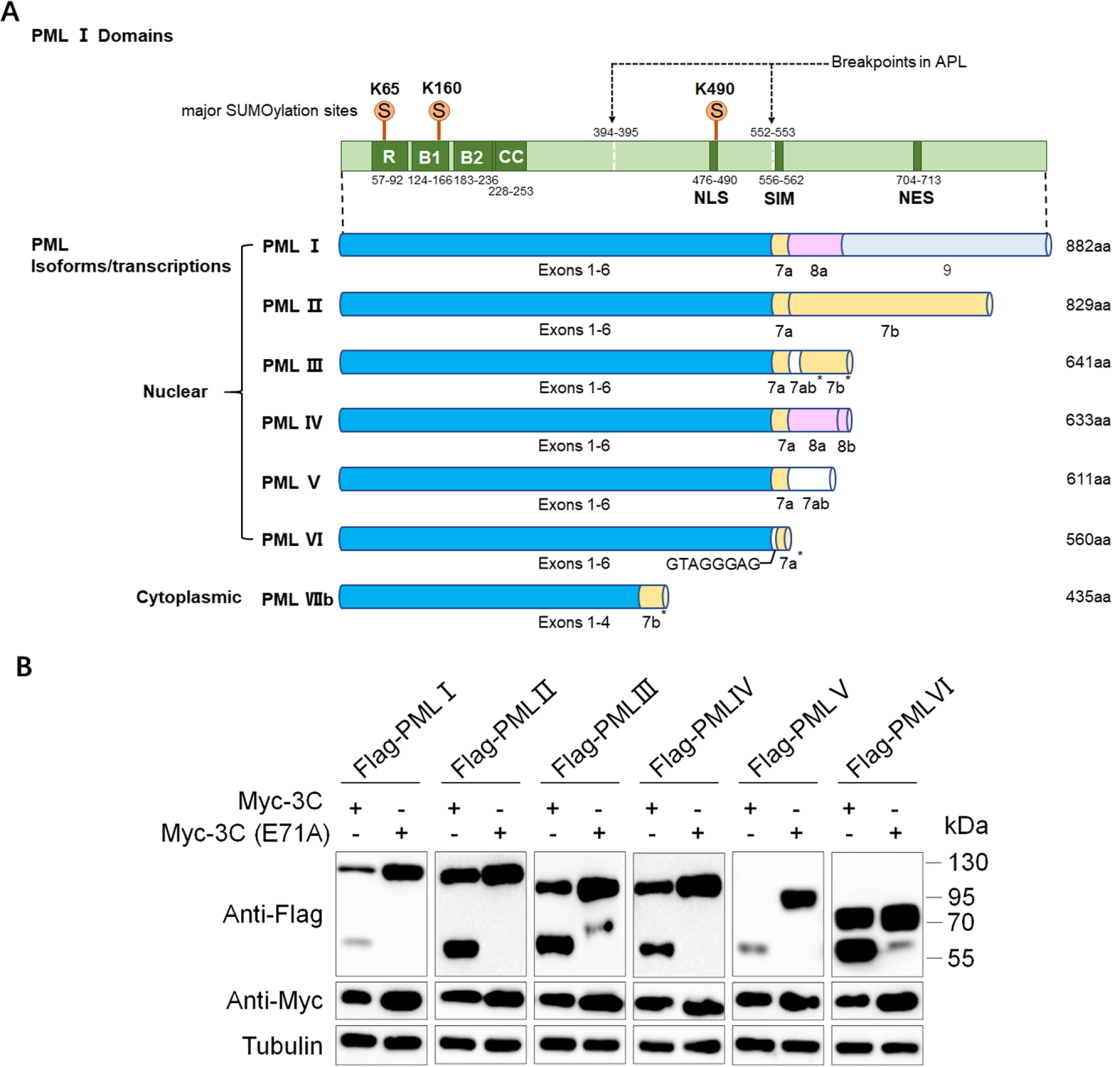
EV71 3C protease cleaves PML. **A** Structure of the PML isoforms. The diagram presents the exon composition of different mRNA variants coding seven PML isoforms (PMLI–VIIb) and the protein length. Light color is used to highlight the difference of their 3′-terminus. The domains, the motifs, and amino acid (aa) positions relative to the PMLI are shown, including RBCC motif, NLS, NES, SIM, two breakpoints in APL, and three major (K65, K160, and K490) SUMOylation sites. **B** Co-transfection analysis of six nuclear isoforms of PML and 3C or 3C (E71A) mutant. Cell lysates of HEK293T were tested by Western blotting after 36 h of transfection

Promyelocytic leukemia (PML) protein, also known as TRIM19, MYL, PP8675, or RNF71, is a member of the tripartite motif (TRIM) protein family [22]. PML was originally found in patients who have acute promyelocytic leukemia (APL), which is caused by the fusion of *PML* with the retinoic acid receptor alpha (*RARA*) gene, which results in the expression of a chimeric protein PML-RARα [23–25]. PML is the primary organizer of PML nuclear bodies (PML-NBs) and plays an important role in genome damage responses, cellular senescence, programmed cell death, and angiogenesis [22]. PML can also be induced by both IFN-I and IFN-II, suggesting that PML could be part of the intrinsic and innate immunity [26]. There are seven isoforms, PMLI–VIIb, generated by alternative splicing from a single gene [27]. All PML isoforms share an identical N-terminal region, which is called RBCC/TRIM motif consists of a RING motif (R), two additional zinc-finger motifs (B-boxes; B), and a coiled-coil domain (CC), but differ in their C-terminal sequences (Fig. 1A). Specifically, PML I–VI are nuclear isoforms with a nuclear localization signal (NLS), while PMLVIIb is a cytoplasmic isoform. PML isoforms are involved in antiviral activity. A previous study found that decreasing overall PML levels promotes both EV71 viral protein expression and viral replication and that overexpressing the PMLIII and PMLIV isoforms rendered cells resistant to EV71 infection. PML selectively inhibited autophagy in infected cells, which facilitated antiviral action against EV71 infection. In contrast, EV71 infection could disrupt PML-NBs formation and induce degradation of PML protein by EV71 3C protease [28]. However, the underlying mechanism is elusive. This study demonstrated that PML could be cleaved by EV71 3C protease independent of its RNA binding activity and identified the cleavage sites of PMLIV, the isoform that could suppress EV71 replication. This study gives details to reveal EV71 virus-host interaction which is benefit for exploring the pathogenesis of viral diseases and developing potential treatment.

## Material and methods

### Cell culture and virus

HEK293T and HeLa cells were maintained in Dulbecco's modified Eagle's medium (DMEM) (high glucose; Gibco, Grand Island, NY, USA) supplemented with 10% fetal bovine serum (Hyclone, Logan, UT, USA) and 1% penicillin/streptomycin (Gibco, Grand Island, NY, USA). HeLa-Scrambled^KO^ and HeLa-PML^KO^ cells were also cultured in the same medium supplemented with puromycin (5 μg/mL). All cells were cultured at 37 °C in a 5% CO_2_ container. EV71 was recovered from pSVA-EV71 infectious clone (strain SK-EV006/Malaysia/97, Accession code: AB469182.1), gifted by Professor Zhiyong Lou (Tsinghua University, Beijing, China). Briefly, the EV71 infectious clone was linearized with *Sal*I, and the resultant linearized DNA was in vitro transcribed to RNA, which subsequently was transfected into RD cells using Lipofectamine 2000 (Invitrogen) according to the manufacturer’s instructions. Viruses were propagated in RD cells, then the cell supernatants were collected by centrifugation at 3000×*g* for 5 min, and the target viruses were stored at − 80 °C. The titers were measured by median tissue culture infective dose (TCID_50_) assay. To the point, seeded 1 × 10^4^ RD cells per well in 96-well plates; after overnight culture, the viruses were serially diluted 10-fold with DMEM containing 10% FBS (10^–1^- to 10^–8^-fold dilutions) and added to RD cell, the plates were then incubated at 37 °C in 5% CO_2_; cytopathic effects (CPE) was observed under the microscope after 3 to 4 days post infection; calculated the TCID_50_ of EV71 virus. EV71 at the indicated multiplicity of infection (MOI) was incubated with cells in DMEM without FBS for 1 h, and the medium was then replaced with DMEM containing 2% FBS.

### Plasmids and transfection

pRK5-Flag series plasmids (Flag-PMLI–Flag-PMLVI) were kindly provided by Professor Jun Tang (State Key Laboratory of Agrobiotechnology, China Agricultural University, Beijing, China). pRK5-Flag vector plasmid was generated from pRK5-Flag-PMLI in our laboratory. The plasmids expressing Myc-3C (E71A), with impaired proteolytic activity, Myc-3C (R84Q) and Myc-3C (V154Q), with impaired RNA-binding activity, were generated using a QuikChange site-directed mutagenesis kit (Stratagene, La Jolla, CA, USA) based on pQCXIP-Myc-3C, which expressed wildtype EV71 3C protein with a Myc tag on its C-terminal. Briefly, prepared the needed segments and added into the tubes in a certain dose according to the instruction, then run the thermal cycle reaction for 16 cycles, and finally placed the reaction on ice for 2 min to cool the reaction. The vector pQCXIP-Myc was reconstructed from pQCXIP (Clontech) in our laboratory. pRK5-Flag-PMLVI variants (Q430A, Q444A) were also generated by the same method based on pRK5-Flag-PMLVI. pRK5-Flag-PMLVI (Q430A/Q444A) was generated based on pRK5-Flag-PMLVI (Q444A). The sequence of QuikChange Primer used were as follows: 5′-GAACAAGGGGTTAATTTGGCGCTAACCCTAATC, and 5′-GATTAGGGTTAGCGCCAAATTAACCCCTTGTTC for Myc-3C (E71A), 5′-CTAATGAGAAATTCCAAGACATTACTAAGTTC, and 5′-GAACTTAGTAATGTCTTGGAATTTCTCATTAG for Myc-3C (R84Q), 5′-GTGGTGACATCATCTGGAAAGGTCATTG, and 5′-CAATGACCTTTCCAGATGATGTCACCAC for Myc-3C (V154Q), 5′-GTGAAGGCCCAGGTTGCGGCCCTGGGGCTGGCTGAAG, and 5′-CTTCAGCCAGCCCCAGGGCCGCAACCTGGGCCTTCAC for PMLVI (Q430A), 5′-GCCCAGCCTATGGCTGTGGTAGCGTCAGTGCCCGGGGCACACCCCGTG, and 5′-CACGGGGTGTGCCCCGGGCACTGACGCTACCACAGCCATAGGCTGGGC for PMLVI (Q444A). Plasmid transfection was performed using the polyethyleneimine (PEI) reagent (Polyscience, Niles, IL, USA) following the manufacturer's instructions.

### Antibodies and reagents

Anti-Flag, anti-Myc, anti-Tubulin, and secondary antibodies labeled with horseradish peroxidase (HRP) were purchased from Santa Cruz Biotechnology (Santa Cruz, CA, USA). Mouse anti-enterovirus 71 VP1 polyclonal antibody was obtained by immunizing mice using purified VP1 protein induced from *E. coli* BL21 (DE3) transformed with pETH-VP1 recombinant plasmid and was determined by enzyme linked immunosorbent assay (ELISA). The sequence of primer to generate pETH-VP1 were as follows: 5′-CGCGGATCCATGGGAGATAGGGTGGCAGATGT, and 5′-CCGCTCGAGTTAAAGGGTAGTAATGGCAGTACG. MG132 and Chloroquine (CQ) were purchased from Sigma-Aldrich (St. Louis, MO, USA) and then the solution in DMSO. MG-132 was used at 5 μM, and Chloroquine was used at 100 μM.

### Western blotting

Cells were harvested and lysed in radioimmunoprecipitation assay (RIPA) buffer supplemented with protease inhibitor cocktail (Roche, Indianapolis, IN, USA) on ice for 10 min and then heated at 100 °C for 20 min, followed with clearing by centrifugation at 13,000×*g* for 5 min at 4 °C. Cell lysates or immunoprecipitated materials were subjected to SDS-PAGE (10 to 12% polyacrylamide) and then transferred to a polyvinylidene difluoride (PVDF) membrane (GE Healthcare, Chicago, IL, USA). The blotted PVDF membranes were blocked with 5% dry milk for 45 min at room temperature. Cellular or viral proteins were probed with specific primary antibodies followed by horseradish peroxidase (HRP) conjugated immunoglobulins as secondary antibodies. Then, the chemiluminescence signal was detected using Luminata™ western HRP substrate (Millipore, Billerica, MA, USA) and visualized with Junyi Capture e610 System (JUNYI, Beijing, China). Some results were analyzed by Image J (Version 1.52v). In brief, converted the images to gray-scale form; drew rectangular selection to enclose a single lane and repeated to set all the blots rectangles in place; drew a profile plot of each lane and then drew a line across the base of the peak to enclose the peak; highlighted all the peaks and checked the values from the Results window; pasted data into a spreadsheet and calculated the relative density of every blots.

### Coimmunoprecipitation

Transfected cells were lysed with RIPA buffer containing protease inhibitor cocktail (Roche, Indianapolis, IN, USA) on ice for 10 min. Lysates were incubated with indicated antibody (Sigma, St. Louis, MO) in 500 μL RIPA buffer at 4 °C overnight on a rotator in the presence of protein A-agarose beads (Santa Cruz Biotechnology, Santa Cruz, CA, USA). The immunocomplex captured on the protein A-agarose was fractionated by 10% or 12% SDS-PAGE and subjected to Western blotting.

### Immunofluorescence assay

HeLa cells were seeded onto 22 mm diameter coverslips and harvested at 36 h post-transfection. The cells were washed two times with PBS, fixed with 4% paraformaldehyde diluted by PBS at room temperature for 10 min, and permeabilized using 0.1% Triton X-100 for 10 min at room temperature. Then cells were incubated in a blocking solution (5% bovine serum albumin, BSA in PBS) for 1 h at room temperature and washed with PBS. The cells were then incubated at room temperature with anti-Flag (1:200) or anti-Myc (1:200) diluted in PBS containing 0.1% BSA and 0.02% sodium azide for 2 h. After they had been washed three times with PBS containing 0.1% Tween 20® (PBS-T), the cells were incubated with fluorescein (FITC)-conjugated AffiniPure Goat Anti-Mouse IgG (diluted 1:500) or Rhodamine (TRITC)-conjugated AffiniPure Goat Anti-Rabbit IgG (diluted 1:500) for 1 h at room temperature. Finally, the cells were stained with DAPI (diluted 1:2000 in PBS) and mounted with Antifade Mounting Medium (Beyotime, Shanghai, China) for confocal microscopy analysis. The images were captured by Leica TCS SP5 Confocal system, with 488 nm and 550 nm laser lines.

## Results

### EV71 3C protease cleaves PML

Promyelocytic leukemia (PML) protein has seven isoforms, which consist mainly of six nuclear isoforms, including NLS designated PMLI to PMLVI, and one cytoplasmic isoform, the shortest PMLVIIb (Fig. 1A). It has been reported that enterovirus 71 3C protease could mediate PMLIII and IV degradation instead of 2A protease [28]. Considered that EV71 3C protein was mainly functioned as a protease to cleave virus precursor protein and multiple host proteins, we hypothesized that EV71 3C might cleave nuclear PML isoforms which are major organizers of PML-NBs. HEK293T cells were transfected with Flag-PMLI–VI separately along with Myc-3C or Myc-3C (E71A), a mutant that had been proved to abolish the protease activity only. At 36 h after the transfection, the cells were harvested to imply Western blotting assays. As shown in Fig. 1B, compared with 3C (E71A), EV71 3C reduced all the six nuclear isoforms of PML and yield low molecular weight products, approximately 60 kDa, specifically. These results support that the decrease of PML caused by 3C protease cleaved PML.

### EV71 3C protease cleaves specifically

**Fig. 2:**
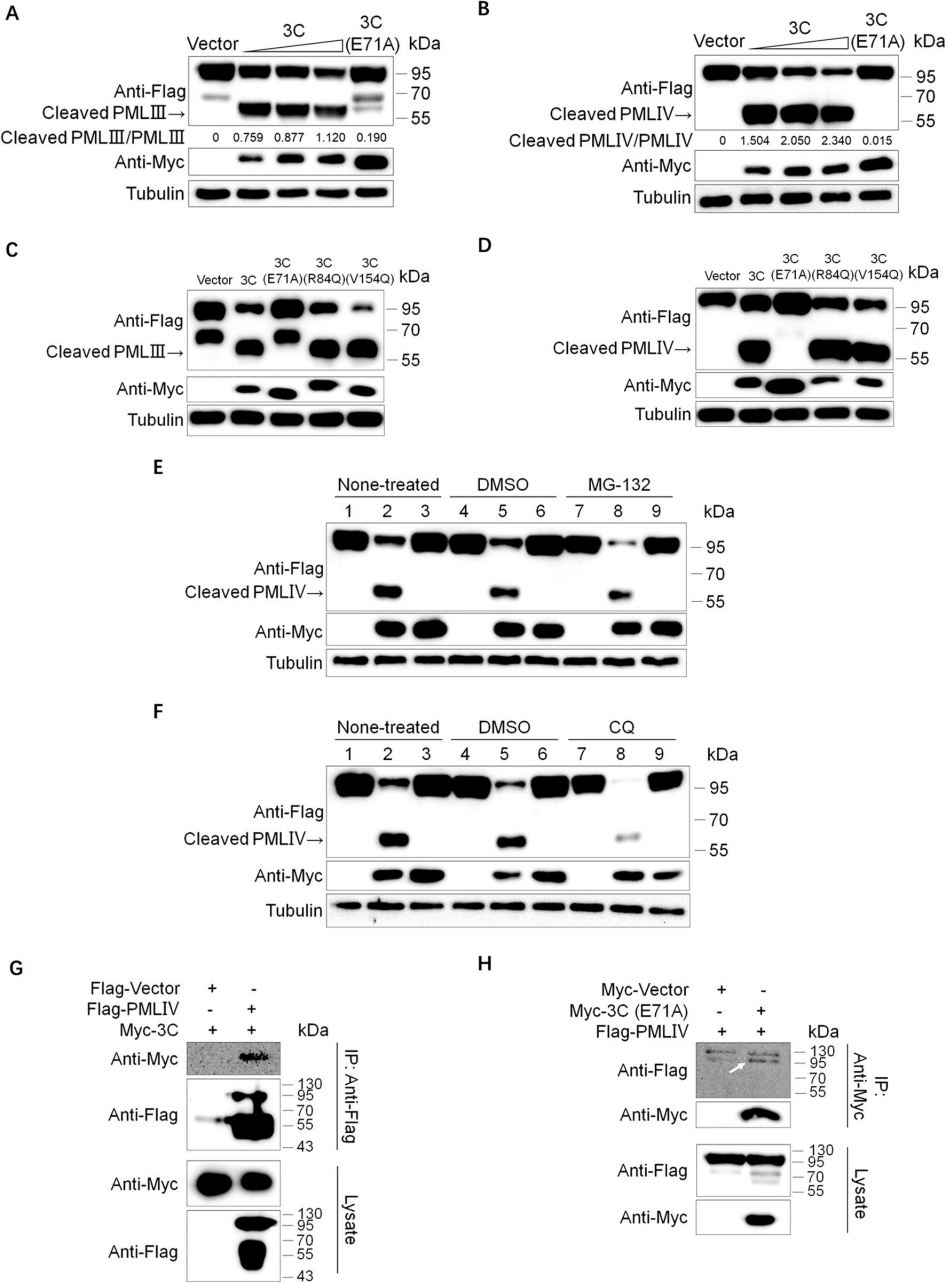
EV71 3C protease cleaves PML specifically. **A**, **B** HEK293T cells were co-transfected with Flag-PMLIII (**A**) or Flag-PMLIV (**B**) and increasing amounts of Myc-3C (0, 0.2, 0.5, and 0.8 μg, respectively) or Myc-3C (E71A) (0.5 μg). After 36 h, cell lysates were harvested and subjected to Western blotting in which anti-Flag, anti-Myc, and tubulin antibodies were used. **C**, **D** Co-transfection analysis of Flag-PMLIII (**C**) or Flag-PMLIV (**D**) and different 3C variants (3C, E71A, R84Q, and V154Q). After 36 h of transfection, cell lysates were tested by Western blotting. **E** HEK293T cells were treated in the presence and absence of 5 μM MG-132 for 5 h after 32 h of co-transfection with Flag-PMLIV and 3C (lane 2, 5, 8) or 3C (E71A) (lane 3, 6, 9) or vectors (lane 1, 4, 7). Cell lysates were immunoblotted with anti-Flag, anti-Myc, or β-actin antibodies. **F** HEK293T cells were treated in the presence and absence of 100 μM Chloroquine (CQ) for 5 h after 32 h of co-transfection with Flag-PMLIV and 3C (lane 2, 5, 8) or 3C (E71A) (lane 3, 6, 9) or vectors (lane 1, 4, 7). Cell lysates were subjected to Western blotting. **G**, **H** HEK293T cells (4 × 10^6^) were co-transfected with empty vector or Flag-PMLIV and Myc-3C (**G**) or pQCXIP-Myc vector or Myc-3C (E71A) and -PMLIV (**H**). Coimmunoprecipitation was performed with rabbit anti-Flag and rabbit anti-Myc antibodies after 36 h of transfection. Samples of both cell lysates and immunoprecipitates were tested by Western blotting and probed with mouse anti-Flag and mouse anti-Myc antibodies. A white arrow was used to highlight the Immunoprecipitation blot. The blots sized approximately 130 kDa were an unspecific band. The key blots were quantified with ImageJ software

Now that EV71 3C might cleave all the six nuclear PML isoforms, we want to determine whether this cleavage function is specific between 3C protease and PML exactly. The previous study has demonstrated that cellular PMLIII and PMLIV could suppress EV71 infection effectively [28]. Therefore, we chose PMLIII and PMLIV and transfected them together with increasing amounts of Myc-3C in HEK293T cells, while 3C E71A mutant was the negative control. It was clear to see that both full-length proteins gradually decreased with the gradual increase of 3C concentration (Fig. 2A, B), which means the cleavage was conducted in a protease dose-dependent manner. 3C protease of enterovirus 71 also possesses RNA-binding activity, and a previous study indicated that mutations in the RNA-binding regions (amino acid positions 82–86 and 154–156) could influence 3C proteolytic activity [15]. So next, we checked whether RNA-binding activity would influence cleavage. The Western blotting assays showed that EV71 3C R84Q and V154Q mutants could still cleave PMLIII and PMLIV, which is distinct from E71A mutant obviously (Fig. 2C, D). This result implied that R84Q and V154Q did not affect protease function, and proteolytic activity but not RNA-binding activity of EV71 3C was vital to PML cleavage.

Since 3C could mediate the reduction of PMLIV dramatically, we wondered whether the proteasome pathway and lysosomal pathway participated in this degradation. Still, HEK293T cells were transfected with PMLIV and Myc-3C, Myc-3C (E71A), or vector. Different from the former experiments, MG-132 (proteasome/calpain inhibitor) and Chloroquine (CQ, autophagy-lysosome inhibitor) were applied as the experimental group. DMSO was the organic dissolvent of the two drugs and was used as another negative control. From Fig. 2E, F, we observed that neither MG-132 nor CQ could impair the cleavage because the reduction of PMLIV and ~ 60 kDa products still existed. Last, we confirmed the interaction of EV71 3C protease and PMLIV in host cells by transfection of HEK293T cells with Myc-3C or Myc-3C (E71A) and Flag-PMLIV, followed by coimmunoprecipitation with an anti-Flag or anti-Myc antibody. The 3C protease was coimmunoprecipitated with PMLIV and vice versa (Fig. 2G, H). Altogether, EV71 3C protease cleaved PMLIV directly and, specifically, irrelevant of its RNA-binding activity and proteasome or lysosomal pathway in host cells.

### The cleavage sites of 3C protease are located at Q430–A431 and Q444–S445 in PMLIV

**Fig. 3:**
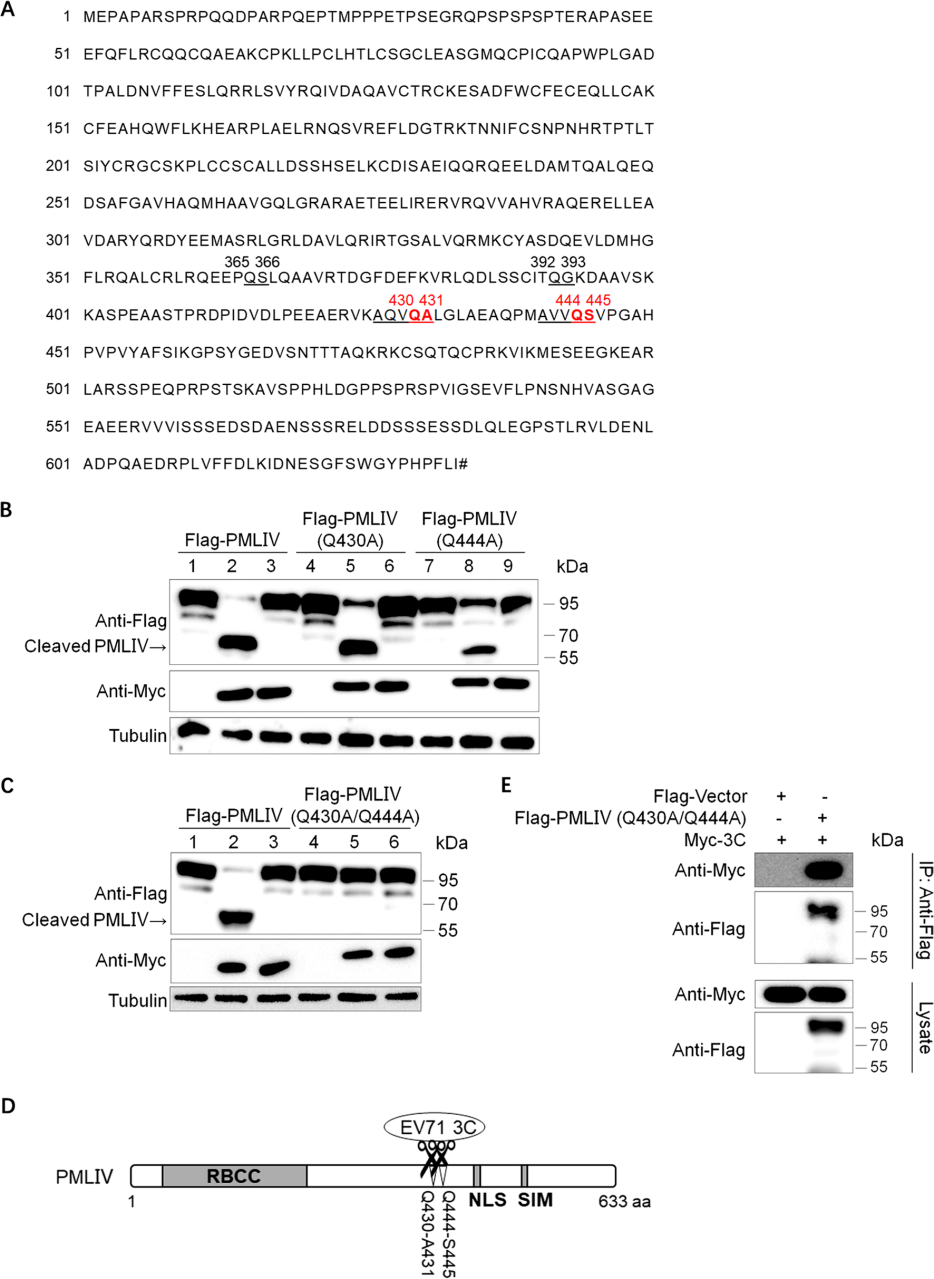
Q430–A431 and Q444–S445 are the sites of PMLIV cleavage by 3C. **A** The amino acids sequence of PMLIV protein (NCBI Reference Sequence: NP_002666.1). The predicted cleavage sites of EV71 3C protease were marked in red. The Hash sign represents the terminator. **B** Co-transfection analysis of PMLIV variants and 3C (lane 2, 5, 8) or 3C (E71A) (lane 3, 6, 9) or vectors (lane 1, 4, 7) in HEK293T cells. After 36 h, lysates were subjected to Western blotting. **C** Co-transfection analysis of PMLIV (Q430A/Q444A) dual-mutation variant and 3C (lane 2, 5) or 3C (E71A) (lane 3, 6) or vectors (lane 1, 4) in HEK293T cells. After 36 h, lysates were tested by Western blotting. **D** Schematic demonstration of the identified cleavage sites of 3C in PMLIV. **E** HEK293T cells were transfected with Myc-3C and/or Flag-PMLIV (Q430A/Q444A) as indicated for 36 h. The cell lysates were subjected to Flag IP, and samples of both cell lysates and immunoprecipitates were tested with the indicated antibodies

To clarify the cleavage specificity, we identified the definite cleavage site(s) of EV71 3C in PMLIV. Firstly, we analyzed the potential site(s) according to its amino acid sequence (NP_002666.1) (Fig. 3A). As shown in the cleavage assay earlier (Fig. 1B), all six PML nuclear isoforms share the same cleaved products with an approximate molecular weight of 60 kDa, indicating that the cleavage site(s) might be located in the conservative regions of PML. We also noticed PMLIV was composed of 634 amino acids and the size of the cleavage was about two-thirds of the total molecular weight. Therefore, we predict the site(s) was located between 400aa–440aa. On the other hand, enterovirus 3C protease had Q–X (X was mostly referenced to G or S or A) sequence preference according to previous research [29], and we found Q430–A431 and Q444–S445 in the predicted area. The pRK5-Flag-PMLIV (Q430A) mutant and pRK5-Flag-PMLIV (Q444A) mutant were constructed to characterize these two amino acids. HEK293T cells were transfected with PMLIV wild type or mutants along with Myc-3C or Myc-3C (E71A) for 36 h. The Western blotting result was shown in Fig. 3B, and we surprisingly found that both were still be cleaved by EV71 3C protease. However, either of the PMLIV mutants could not be cleaved completely. Then we postulated logically that both sites were responsible for 3C protease cleavage. The pRK5-Flag-PMLIV (Q430A/Q444A) dual-mutation variant plasmid was constructed and transfected with Myc-3C or Myc-3C (E71A) in HEK293T cells. As demonstrated in Fig. 3C, the cleavage blotting sizing about 60 kDa disappeared, and the PMLIV (Q430A/Q444A) dual-mutation variant was resistant to cleavage totally, illustrating that both Q430–A431 and Q444–S445 were the cleavage sites of 3C in PMLIV (Fig. 3D). This evidence also elucidated why we detected only one cleaved blotting of PMLIV using an anti-Flag antibody. The two cleavage sites are too closed to show only one blotting in the Western blotting assay. We also applied coimmunoprecipitation using an anti-Flag antibody and confirmed that amino acid mutation could not affect the interaction between 3C protease and PMLIV (Fig. 3E). Collectively, these results suggested 3C protease cleaved PMLIV at Q430–A431 and Q444–S445 sites.

### Cleavage of PMLIV impairs PML-NBs production and augments EV71 replication

**Fig. 4:**
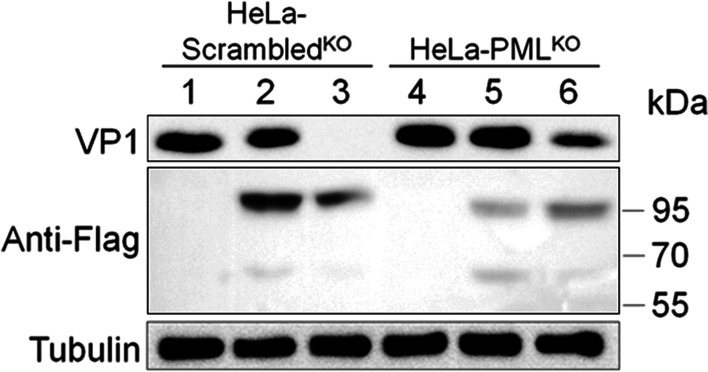
EV71 3C protease cleaves PML to maintain virus replication. The control (HeLa-Scrambled^KO^), and the stable PML-knockout cells (HeLa-PML^KO^) were cultured in 12-well plates to 60% confluency and transfected with Flag-PMLIV (lane 2, 5), Flag-PMLIV (Q430A/Q444A) (lane 3, 6), or vectors (lane 1, 4). After 24 h, all cells were infected with EV71 at the multiplicity of infection (MOI) of 1 for 8 h. EV71 capsid protein VP1, and PMLIV or its variant were analyzed by Western blotting. Tubulin was used as the protein loading control

To further investigate the impact of PMLIV cleavage by EV71 3C protease, we set virus infection experiment. The wild-type PMLIV or Q430A/Q444A dual-mutation variant was transfected into PML knockout cell lines (HeLa-PML^KO^) and non-knockout cell lines (HeLa-Scrambled^KO^). Vector was transfected as control. 24 h after transfection, all cells were infected with EV71 at the multiplicity of infection (MOI) of 1 for 8 h. Then Western blotting was applied using an anti-VP1 antibody to assess virus replication. As shown in Fig. 4, at the same conditions, knockout of PML renders the cells more susceptible to EV71 infection (VP1 blot band in lane 1 was bolder than lane 4, which was the same condition in lane 2 and5, 3 and 6). Meanwhile, EV71 replication had a lower level in PMLIV dual-mutation-variant-expressed cells than wildtype-expressed cells (comparing VP1 blot bands in lane 3 and 2, 6 and 5), suggesting that 3C protease cleaved PMLIV to antagonize its antiviral activity in favor of viral replication; however, 3C could not cleave PMLIV dual-mutation variant, which had stronger antiviral potential.

**Fig. 5:**
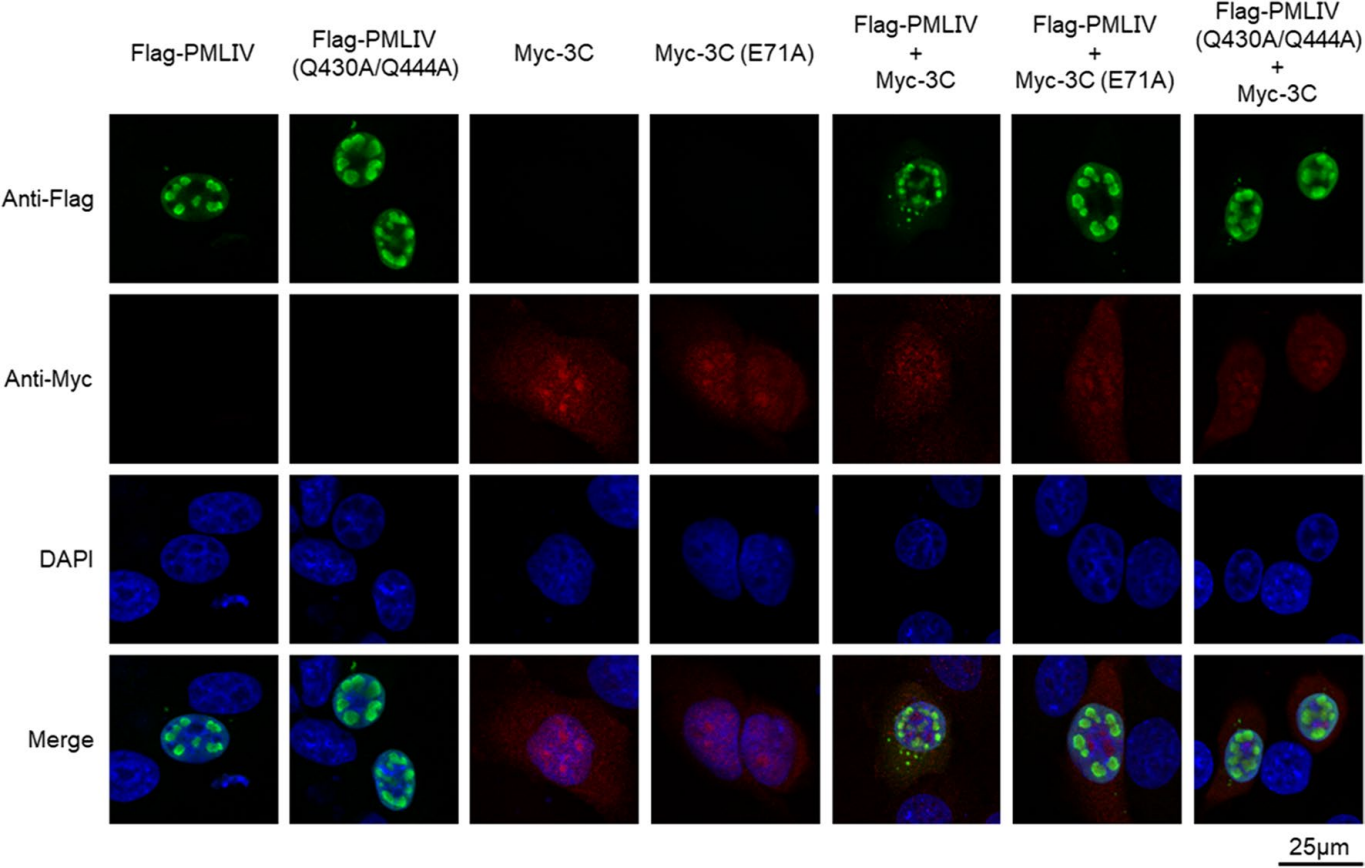
The morphological change of PML-NBs caused by EV71 3C cleavage. HeLa cells were transfected with Flag-PMLIV, Flag-PMLIV (Q430A/Q444A), Myc-3C, Myc-3C (E71A), or co-transfected with their combinations, respectively, for 48 h. Cells were subjected to immunofluorescence staining using an anti-Flag antibody (FITC) or anti-Myc antibody (TRITC), and the cell nuclei were stained with DAPI. Fluorescence images were taken by a Leica TCS SP5 Confocal system

Given that PML functions as a major organizer of PML nuclear bodies (NBs) in most mammalian cells and plays an important role in antiviral activities [30], we applied immunofluorescence analysis to inspect the interfere of 3C protease to PML-NBs directly (Fig. 5). Both PMLIV and PMLIV (Q430A/Q444A) dual-mutant could form normal PML-NBs. While, EV71 3C protein and its mutant were localized in both nucleus and cytoplasm, but more in the nucleus with forming of dots. When 3C protease and PMLIV were co-transfected in HeLa cells, the large clustered spots turned into smaller dots which means PML-NBs were disintegrated by 3C protease. However, this phenomenon was not observed when we co-transfected either PMLIV and 3C (E71A) or PMLIV (Q430A/Q444A) dual-mutation variant and 3C. Overall, our findings suggest that 3C might impair PML-NBs production via PMLIV cleavage and thereby counter its antiviral activities.

## Discussion

3C protein, also known as 3C protease, as one of the seven non-structure proteins of enterovirus 71, is instrumental to viral protein maturation and virus-host interaction. Many host proteins were shown to be cleaved by 3C protease, including hnRNPA1, TAK1/TAB1/TAB2/TAB3 Complex, and PinX1 [17, 31, 32]. This study demonstrated that promyelocytic leukemia (PML) protein is a new substrate cleaved by EV71 3C protease at two sites, Q430–A431 and Q444–S445, and clarified the specific molecular mechanism of PML degradation mediated by 3C protease during EV71 infection. Moreover, our results showed that the degradation was independent of two degradation systems in mammalian cells, autophagy/lysosomal pathway (corresponding inhibitor CQ) and ubiquitin–proteasome pathway (corresponding inhibitor MG132) [33, 34], and the abolition of RNA binding activity did not affect the PML cleavage (Fig. 2C, D, variants Myc-3C (R84Q) and Myc-3C (V154Q)). In addition, ectopic expression of PMLIV mutant that antagonizes 3C protease cleavage could enhance the viral inhibition, proving that 3C cleaves PML to antagonize its antiviral effect during EV71 infection, which supports that targeting of PML-NBs by viral regulatory proteins is a functional strategy to compromise intrinsic antiviral defense and innate immunity [28, 30].

Autophagy and the ubiquitin–proteasome system are the two major pathways to regulate cellular protein degradation [34, 35]. Former studies revealed that HSV-1 and EMCV infections could mediate PML degradation in a proteasome-dependent manner [36–38]. Chen et al*.* found that PML degradation induced by EV71 infection was independent of the proteasome pathway and mediated by 3C^pro^, but not 2A^pro^ [28]. Here, our study further confirmed that 3C mediated PML reduction was not related to proteasome pathway or lysosomal pathway by using MG132 as well as Chloroquine (CQ), but directly cleaved by which PML degradation phenomenon disappeared when single point mutation E71A was introduced in 3C protease core disrupting its cleavage activity. Furthermore, the 3C protease of enterovirus 71 possesses RNA-binding activity, and a previous study indicated mutations in the RNA-binding regions (amino acid positions 82–86 and 154–156) influenced 3C proteolytic activity [15]. However, the 3C V154Q mutant could still cleave PML, indicating that protease activity but not RNA binding activity was responsible for PML cleavage, consistent with the conclusion of cleavage of PinX1 by 3C [17].

Picornaviral 3C protease, reportedly with a wide range of cleavage substrates, plays an important role in the process of picornavirus infection. In addition to helping the virus precursor protein mature, 3C protease can also affect a variety of cellular processes either in cytoplasm or translocate in nucleus, like shutting off cellular gene expression in transcriptional and translational levels, thus promoting virus replication as well as escaping the host's natural immunity [39]. PML, which was confirmed as a novel substrate of EV71 3C protease in this study, is implicated in several cell processes, including antiviral defense. Studies have demonstrated PML possessed antiviral defense against RNA and DNA viruses from different families by stably expressing a single PML isoform or depleting PML by RNA interference in cells [40]. Besides, PML acting as an ISG can be induced by type I and type II IFN, and the increased expression can lead to the high numbers and the bigger size of PML-NBs in the IFN-treated cells. Towards Enterovirus, a study showed that PML isoform III and IV represses EV71 infection partially by inhibiting autophagy in the infected cells [28]. Instead, EV71 3C protease but not 2A protease induced PML degradation, independent of the proteasome pathway. Our study revealed EV71 3C protease cleaved PML to disrupt its antiviral activity. As a result, dual-mutant PML could not be cleaved by 3C protease and showed stronger repression ability than wild type in the ectopic expression cells. All of these results offered new evidence of EV71 evading host intrinsic immunity.

The sequence preference of picornaviral 3C protease cleaved sites has not been investigated comprehensively. It is generally accepted that most 3C proteases preferentially cleave polypeptide with P1-Gln/P1’-Gly (Q–G) junctions [41]. Except for FMDV 3C, other picornaviruses have less strict cleavage, with a small residue at P1', such as Gly (G), Ser (S), and Ala (A) or a hydrophobic residue at P4. Previous studies show that the cleavage site for protease 3C of enteroviruses is often between Q and G or Q and S in the target sequence of AXXQG or AXXQS [29]. In our study, mutational experiments demonstrated that EV71 3C cleaved PMLIV between Q430–A431 junction and Q444–S445 junction simultaneously, and PMLIV Q430A/Q444A dual-mutations abolished cleavage accordingly. Sequence analysis of PMLIV amino acids showed that Q430 and Q444 are in the sequence of 427AQVQA431 and 441AVVQS445, respectively, with the AXXQ(G/S) sequence preference of EV71 3C protein cleavage, and both are in accord with P1-Q/P1'-G/S/A sequence preference of picornaviral 3C protease, which also explains why we found that EV71 3C protease did not cleave between Q365–S366 junction or Q392–G393 junction which seems like the potential cleavage sites. From another perspective, the two cleavage sites confirmed in our investigation locate in an adjacent region of PMLIV nuclear localization sequence (NLS), and, which is reasonable to extrapolate, it is not easy to be obscured conformationally so 3C protease can identify and catalyze to cleavage PML more easily. Of course, solid evidence from complex protein crystallographic analysis is needed to support this view. The interaction of PML with certain viral proteins or with the PML-RARa chimeric protein causes PML-NBs disruption and abolishes normal PML-NBs functions [42]. Furthermore, it is logical to predict that PML protein lost nuclear entry ability without NLS, and its RBCC/TRIM motif is still complete after cleavage and could not form PML-NBs to regulate cell life activity. EV71 3C protease cleavage sites presenting in the conserved region of PMLI–VI protein provide a wide range of antagonism, which might be the result of natural selection between virus and host.

## Conclusion

In summary, we conform that promyelocytic leukemia protein, especially PMLIV, which sequesters viral proteins in PML-NBs and inhibits viral production, is a novel target of EV71 3C cleavage. This cleavage promotes EV71 viral replication. Our study gives a clear clue to reveal virus-host interaction is essential for understanding the pathogenesis of viral diseases and developing potential treatment.

## Data Availability

The datasets supporting the conclusions of this article are included in the article.

## Abbreviations

APL: Acute promyelocytic leukemia
CPE: Cytopathic effect
CQ: Chloroquine
DMEM: Dulbecco's Modified Eagle Medium
EV71: Human enterovirus 71
HFMD: Hand-Foot-and-Mouth Diseases
NLS: Nuclear localization signal
ORF: Open reading frame
PML: Promyelocytic leukemia
PML-NBs: Promyelocytic leukemia nuclear bodies
RARA: Retinoic acid receptor alpha
TRIM: Tripartite motif proteins
